# Pharmacologic screen identifies active combinations with BET inhibitors and LRRK2 as a novel putative target in lymphoma

**DOI:** 10.1002/jha2.535

**Published:** 2022-07-27

**Authors:** Filippo Spriano, Giulio Sartori, Chiara Tarantelli, Marilia Barreca, Gaetanina Golino, Andrea Rinaldi, Sara Napoli, Michele Mascia, Lorenzo Scalise, Alberto J. Arribas, Luciano Cascione, Emanuele Zucca, Anastasios Stathis, Eugenio Gaudio, Francesco Bertoni

**Affiliations:** ^1^ Institute of Oncology Research Faculty of Biomedical Sciences Università della Svizzera Italiana Bellinzona Switzerland; ^2^ Department of Biological Chemical and Pharmaceutical Sciences and Technologies (STEBICEF) University of Palermo Palermo Italy; ^3^ SIB Swiss Institute of Bioinformatics Lausanne Switzerland; ^4^ Department of Oncology Oncology Institute of Southern Switzerland Ente Ospedaliero Cantonale Bellinzona Switzerland; ^5^ Faculty of Biomedical Sciences Università della Svizzera Italiana Lugano Switzerland

**Keywords:** BET, HDAC, JAK, LRRK2, LYMPHOMAS

## Abstract

Inhibitors of the Bromo‐ and Extra‐Terminal domain (BET) family proteins have strong preclinical antitumor activity in multiple tumor models, including lymphomas. Limited single‐agent activity has been reported in the clinical setting. Here, we have performed a pharmacological screening to identify compounds that can increase the antitumor activity of BET inhibitors in lymphomas.

The germinal center B‐cell like diffuse large B‐cell lymphoma (DLBCL) cell lines OCI‐LY‐19 and WSU‐DLCL2 were exposed to 348 compounds given as single agents at two different concentrations and in combination with the BET inhibitor birabresib. The combination partners included small molecules targeting important biologic pathways such as PI3K/AKT/MAPK signaling and apoptosis, approved anticancer agents, kinase inhibitors, epigenetic compounds. The screening identified a series of compounds leading to a stronger antiproliferative activity when given in combination than as single agents: the histone deacetylase (HDAC) inhibitors panobinostat and dacinostat, the mTOR (mechanistic target of rapamycin) inhibitor everolimus, the ABL/SRC (ABL proto‐oncogene/SRC proto oncogene) inhibitor dasatinib, the AKT1/2/3 inhibitor MK‐2206, the JAK2 inhibitor TG101209. The novel finding was the benefit given by the addition of the LRRK2 inhibitor LRRK2‐IN‐1, which was validated in vitro and in vivo. Genetic silencing demonstrated that LRRK2 sustains the proliferation of lymphoma cells, a finding paired with the association between high expression levels and inferior outcome in DLBCL patients.

We identified combinations that can improve the response to BET inhibitors in lymphomas, and LRRK2 as a gene essential for lymphomas and as putative novel target for this type of tumors.

## 1 INTRODUCTION

The covalent addition of acetyl groups to lysins is a histone modification, fundamental for chromatin remodeling and transcription activation [1, 2]. While histone acetyltransferases introduce the acetylation, the Bromo‐ and Extra‐Terminal domain (BET) family proteins BRD2, BRD3, BRD4 and BRDT (Bromodomain testis‐specific protein) recognize and bind to the acetylated histones [1, 2]. The binding is crucial for the recruitment of multiprotein complexes that allow the transcription of genes. The possibility of inhibiting BET proteins has been firstly explored, with good results, in NUT midline carcinoma (NMC), a rare subtype of squamous carcinoma characterized by the fusion oncoprotein BRD4‐NUT [1, 2]. Subsequently, results obtained via genetic and chemical inhibition of BET proteins have shown that targeting this class of proteins has an anticancer effect in multiple tumor models beyond NMC [1, 2]. Preclinical [1, 3‐9] data show that diffuse large B‐cell lymphomas (DLBCL), and in particular the activated B‐cell like (ABC) subtype, are sensitive to the treatment with BET inhibitors, although the clinical activity [1, 10‐13] has been so far rather limited. Here, we have performed a pharmacological screen to identify compounds that can increase the antitumor activity of BET inhibitors in lymphoma. We used as starting model the germinal center B‐cell like (GCB) DLBCL, which shows less sensitivity toward BET inhibitors than ABC type [5, 7, 8]

## 2 MATERIALS AND METHODS

### 2.1 Cell lines

Lymphoma cell lines were cultured according to the recommended conditions, as previously reported [14, 15]. All media were supplemented with fetal bovine serum (10%), Penicillin‐Streptomycin 100X (Euroclone, ECB3001D). Cell line's identity was confirmed by short tandem repeat (STR) DNA fingerprinting using the Promega GenePrint 10 System kit (B9510) [9], and all the experiments were performed within 1 month from being thawed. Cells were periodically tested to confirm mycoplasma negativity using the MycoAlert Mycoplasma Detection Kit (Lonza, Visp, Switzerland).

### 2.2 Pharmacological combination screening

Cells were exposed to birabresib in combination with 348 compounds from a selleckchem custom library composed of FDA (Food and Drug Administration) approved compounds or small molecules targeting important pathways in lymphoma. The list of the 384 compounds is available in Table S1. Cells were seeded at 10,000 cell/well and treated with two different concentrations (1µM and 20 nM) in single or combination with birabresib (100 nM) for 72 h. After 72 h MTT (3‐[4,5‐dimethylthiazol‐2‐yl]‐2,5‐diphenyltetrazolium bromide) test was performed. Compounds of interest for further studies were defined as the ones resulting in a 1.5‐fold decreased proliferation with the combination compared to the single compounds. Compounds giving, already as single treatment, less than 30% of proliferating cells at a specific concentration were excluded from the combo analysis.

Selected compounds were then validated in dose response combination treatments (8 × 8) as previously described [14]. Briefly, compounds were given at concentrations from 1500 to 22 nM, 1:2 dilutions. MTT and IC50 calculation were done as previously described [15]. For IC_50_s above 1 µM, they were calculated interpolating all points to construct a curve until the 50% of living cells. All compounds were purchased from Selleckchem (TX, USA).

Synergy was assessed with Chou‐Talalay Combination Index (CI): synergism (<0.9), additive (0.9–1.1), antagonism/no benefit (>1.1).

### 2.3 In vivo experiment

Mice maintenance and animal experiments were performed under the institutional guidelines established for the Animal Facility and with study protocols approved by the local Cantonal Veterinary Authority (license TI‐22‐2015). NOD‐Scid mice were obtained from The Harlan Laboratory (S. Pietro al Natisone, Udine, IT). Xenografts were established by injecting WSU‐DLCL2 lymphoma cells (15 × 10^6^ cells/mouse, 100 µl of PBS) into the left flanks of female NOD‐Scid mice (6 weeks of age, approximately 20 gr of body weight). Tumor size was measured on regular basis and until tumors reached around 5 mm in diameter (100 mm^3^). Tumor size was measured using a digital caliper (tumor volume [mm^3^] = D×d^2^/2). Mice were treated with vehicle (30% PEG400 in water, P.O.), birabresib and LRRK2‐IN‐1 both at 100 mg/kg (once daily; 5 days on/2 days off) and combination of each other. Differences in tumor volumes were calculated using the nonparametric Mann–Whitney test (GraphPad Software, Version 9.3.1). The *p*‐value (P) for significance was <0.05. The coefficient of drug interaction (CDI) is calculated as follows: CDI  =  AB/(A × B). AB is the ratio of the tumor volume (mm^3^) combination groups to control group. A or B is the ratio of the single agent group to control group. Thus, CDI < 1 synergistic effect, CDI = 1 additive effect, CDI > 1 no benefit.

### 2.4 Gene silencing

For transient knock down, we used the Amaxa 4D Nucleofector system (Lonza) to introduce four LRRK2 siRNAs from ON‐TARGET SMARTpool siRNA or a nontargeting siRNA as control (Dharmacon GE Healthcare now Horizon Discovery Ltd.). Protocols were followed according to the SG Cell Line 4D‐Nucleofector X Kit L (Lonza). In brief, 2 × 10^6^ cells were prepared and resuspended in 100 µl SG solution with 800 pmol siRNA. Efficiency was confirmed 48 h after nucleofection by flow cytometry and cells were harvested for protein lysates.

### 2.5 Immunoblotting analyses

Protein extraction, separation, and immunoblotting were performed as previously described [15]. The following antibodies were used: anti‐LRRK2 (Ab 133474) from Abcam, anti‐AKT (CST 9272), anti‐p‐AKT (Ser 473) (CST 4060), anti‐GSK3β (CST 9832), anti‐GSK3β (Ser 9) (CST 9322) from Cell Signaling Technology, anti‐GAPDH (FF26A) from eBioscience, secondary mouse (NA931V) and rabbit (NA934V) antibodies from GE healthcare. Data were analyzed with Fusion solo software (Vilberg, France). Densitometry data were z‐score transformed, and one tail t‐test was applied.

### 2.6 Survival and expression analysis in clinical specimens

Two publicly available datasets of DLBCL clinical specimens (phs001444.v2.p1 [16], EGAD00001003140 [17]) were processed as previously described [18, 19]. We investigated the impact of LRRK2 mRNA level on overall survival, and the survminer package in R was used to identify the optimal cutoff point for high‐ and low‐LRRK2 expressers (7.79 lcpm, EGAD00001003140; [17] 9.625 log2 FPKM, phs001444.v2.p1 [16],) in terms of different overall survival. The Kaplan–Meier method and the log‐rank test were performed to study the ability of the LRRK2‐induced classification to predict different survival probability. Cox univariate and multivariate analyses were performed without violating the proportional hazards.

## 3 RESULTS

### 3.1 Pharmacological screening identifies active combinations with BET inhibitors

**FIGURE 1:**
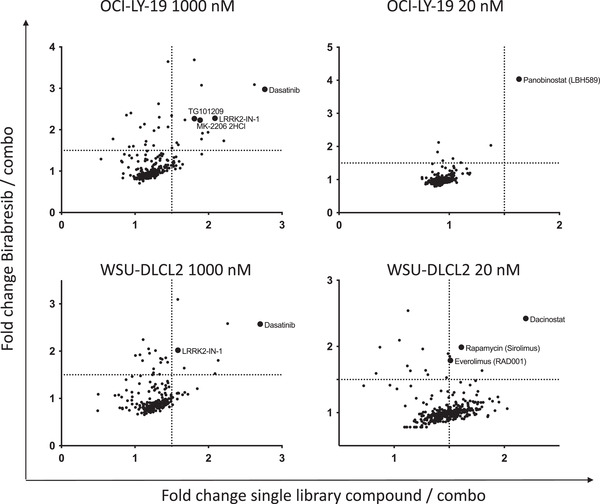
Chemical screening of potential combinatorial partners for birabresib in WSU‐DLCL2 and OCI‐LY‐19 GCB diffuse large B‐cell lymphoma (DLBCL) cell lines. Three hundred forty‐eight compounds were administered to cells as single agents (20 nM, 1000 nM) and in combination with birabresib (100 nM). After 72 h, MTT test was performed. Compounds giving a 1.5‐fold decreased proliferation with the combination than with the individual compounds were further investigated

To identify novel and potentially active combinations with BET inhibitors, the GCB DLBCL cell lines OCI‐LY‐19 and WSU‐DLCL2 were exposed to 348 compounds given as single agents (20 nM, 1 µM) and in combination with birabresib, at a concentration lower than the calculated IC_50_, (100 nM) (Figure 1; Table S1). The combination partners included approved kinase inhibitors, epigenetic compounds and small molecules targeting important biologic pathways, such as PI3K/AKT/MAPK signaling and apoptosis, as listed in Table S1. The screening identified a series of compounds leading to a stronger antiproliferative activity when given in combination than as single agents. The potentially active combination partners included the HDAC inhibitors panobinostat and dacinostat, the mTOR inhibitor everolimus, the ABL/SRC inhibitor dasatinib, the AKT1/2/3 inhibitor MK‐2206, the JAK2/BET inhibitor TG101209 and the LRRK2 inhibitor LRRK2‐IN‐1.

**FIGURE 2:**
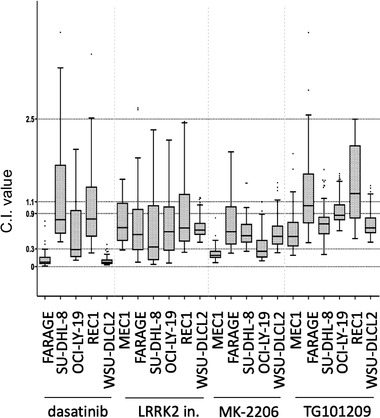
Combinations of birabresib with identified inhibitors in lymphoma cell lines. Box‐plots of the combination index (CI) values obtained in individual cell lines. Y‐axis: CI values. In each box‐plot, the line in the middle of the box represents the median CI value for the different concentrations combined. The box extends from the 25th to the 75th percentile (interquartile range, IQ); the whiskers extend to the upper and lower adjacent values (i.e., ±1.5 IQ); outside values have been omitted from the figure. CIs for birabresib/dasatinib in MEC1 and birabresib/MK‐2206 in REC1 were not plotted due to median value >3

Since synergism of BET inhibitors with HDAC and mTOR inhibitors has already been described, even in DLBCL cell lines [1, 4, 5, 7], we focused on the other combinations. Following validation of the results obtained by the screening in the same two cell lines initially used, we tested the combinations also in two additional GCB DLBCL cell lines (SU‐DHL‐8 and FARAGE), one mantle cell lymphoma (MCL) (REC1) and one chronic lymphocytic leukemia (CLL) cell line (MEC1) (Figure 2). The combination of LRKK2‐IN‐1 was synergistic with birabresib in all the six cell lines and with pelabresib in 4/6 cell lines. The AKT1/2/3 inhibitor MK‐2206 in combination with birabresib or with pelabresib was synergistic in 5/6 cell lines, using the Chou‐Talalay CI [20]. Dasatinib in combination with birabresib or with pelabresib was synergistic in 5/6 cell lines. The JAK2 inhibitor TG101209 in combination with birabresib or with pelabresib was synergistic in 4/6 and 3/6 cell lines, respectively.

### 3.2 In vitro efficacy of the combination of birabresib with LRRK2 inhbitors

Since the most novel finding was the synergism with LRRK2 inhibitor, we focused on this combination. The combination between LRRK2‐IN‐1 and birabresib was also evaluated using the efficacy and potency parameter (CIT) according to the MuSyC algorithm [21], showing additivity in efficacy and between additivity and synergism in the potency parameter in all the cell line tested (Figure S1).

To extend the findings beyond birabresib, we also used pelabresib (CPI‐0610), a BET inhibitor currently in phase 3 trial for myelofibrosis (NCT04603495), to validate the synergism with LRRK2‐IN‐1 (Figure S2).

Birabresib showed synergism also with two other LRRK2 inhibitors (PF‐06447475 and GNE‐0877) with an activity superior to the predicted for additivity response (Figure S3).

The in vitro combination between LRRK2‐IN and birabresib significantly increased the induction of apoptosis compared to single treatments in WSU‐DLCL2 and with a lesser extent in OCI‐LY‐19 (Figure S4). Cell lines were treated with two concentrations of birabresib (100, 500 nM) and two concentrations of LRRK2 inhibitor (1, 2 µM) close to the IC_50_s in single and combination for 72 h followed by annexin V assay.

### 3.3 In vivo efficacy of the combination of birabresib with LRRK2‐IN‐1

**FIGURE 3:**
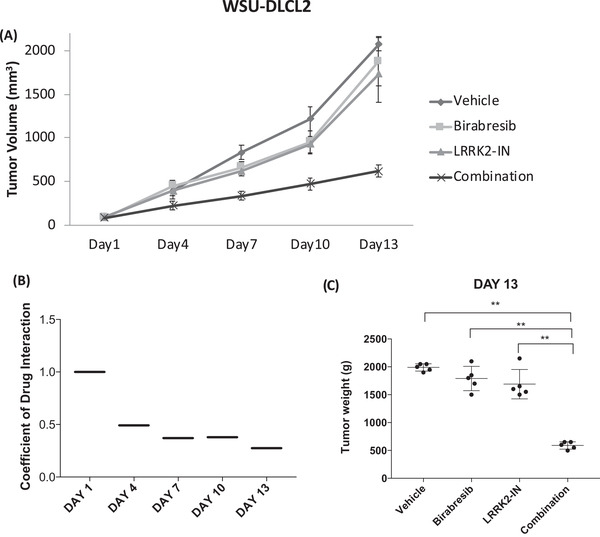
Birabresib combined with LRRK2‐IN‐1 show stronger antitumor activity rather than the single agents in WSU‐DLCL2 GCB diffuse large B‐cell lymphoma (DLBCL) model. (A) Treatment with birabresib (100 mg/kg, P.O. 5 days ON/week), LRRK2‐IN‐1 (100 mg/kg, P.O. 5 days ON/week), their combination or vehicle started when tumors became visible (>80mm ^3^). Y‐axis, tumor volume in mm^3^ (mean ± standard deviation). X‐axis, days of treatment. (B) Coefficient of Drug Interaction (CDI); CDI < 1 synergistic effect, CDI = 1 additive effect, CDI > 1 no benefit. (C) Tumor weight at the end of the experiment (DAY 13). **p value <0.01 calculated with nonparametric Mann–Whitney test

The combination of birabresib with LRKK2‐IN‐1 was validated using an in vivo GCB DLBCL model, the WSU‐DLCL2 cell line. Mice were divided into four groups of five animals each and were treated with birabresib (100 mg/kg po, 5 days ON/week), or LRKK2‐IN‐1 (100 mg/kg po, 5 days ON/week), or birabresib plus LRKK2‐IN‐1 (same schedule as single agents), or with vehicle only. The combination of birabresib with the LRRK2 inhibitor LRKK2‐IN‐1 showed superiority than single treatments in delaying tumor growth (*p* < 0.001) on days 4, 7, 10, and 13 (Figure 3). The CDI showed synergism (CDI < 1 synergistic effect) between the two compounds starting from day 4 and for the entire duration of the treatment. At the end of the treatment (day 13), all mice were sacrificed, and the weights of tumors treated with the combination were significantly lower than single treated mice.

Both single treatments and the combination did not cause any body weight loss, and all the mice were well body conditioned (BC3) [22].

### 3.4 LRRK2 has a prognostic role in DLBCL clinical specimens

LRRK2 is a kinase regulating the WNT [23], MAPK [24], and MTOR [25] signaling pathways, and it is involved in the regulation of cell proliferation, apoptosis, inflammation [26], and autophagy [25].

Given that very little is known about LRRK2 in lymphomas, we looked at its expression pattern in DLBCL, taking advantage of two available datasets (phs001444.v2.p1 [16], EGAD00001003140) [17]. LRRK2 was expressed at higher levels in ABC than in GCB DLBCL (Figure S5A).

Also, high expression levels were associated with inferior outcome. In the first dataset [16], by cox univariate analysis, the hazard ratio was ∼2.12 (*p* = 0.023) for the high compared to the low LRRK2 expressors, while in the second dataset [17], it was ∼2.14 (*p* = 0.032) (Figure S5B). By multivariate analysis, the prognostic impact was independent of the cell of origin (COO) (*p* = 0.039) and International Prognostic Index (IPI) (*p* = 0.02624) in the first [16] but not in the second dataset [17], (*p* = 0.28 and *p* = 0.102, respectively, for COO and IPI).

### 3.5 Genetic and pharmacological inhibition of LRRK2 is toxic for DLBCL cells

**FIGURE 4:**
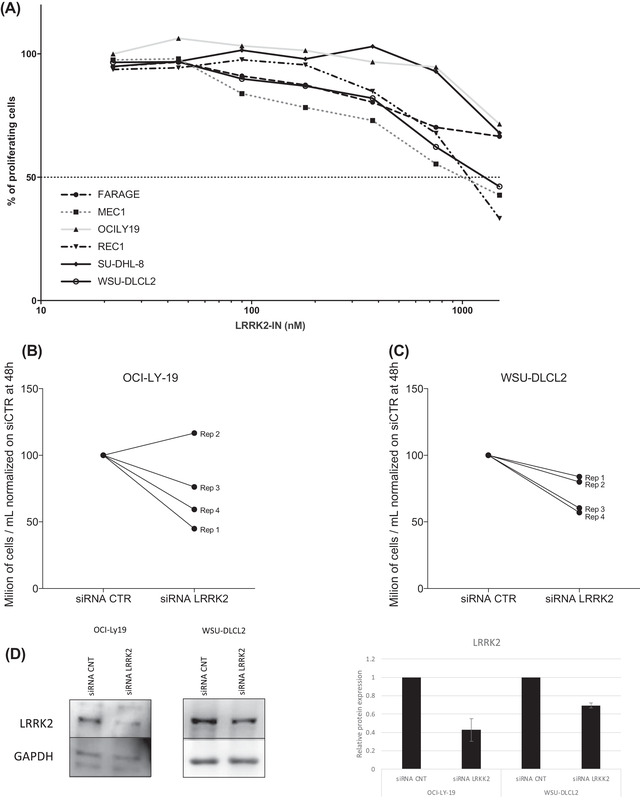
*LRRK2* is important for lymphoma cell lines. (A) Lymphoma cell lines treated with increasing doses of LRRK2‐IN‐1 for 72 h. (B) Cells treated with pool of siRNAs targeting *LRRK2* at 500 nM for 48 h showed decreased cell growth in OCI‐LY‐19 and (C) in WSU‐DLCL2. Rep1, Rep2, Rep3, Rep4 = Replicate 1, 2, 3, 4. (D) Representative immunoblot, of two replicates, performed after 48 h of LRRK2 siRNAs with its quantification. The quantification is represented as relative protein expression of LRRK2 to siRNAs control and normalized to the respective housekeeping GAPDH

We treated four GCB DLBCL (FARAGE, OCI‐LY‐19, SU‐DHL‐8, and WSU‐DLCL2), one MCL (REC1), and one CLL (MEC1) cell lines with LRRK2‐IN‐1 as single agent (Figure 4A). The LRRK2 inhibitor showed a median IC_50_ of 1.6 µM (MEC1, IC_50_ = 1 µM; REC1, IC_50_ = 1 µM; WSU‐DLCL2, IC_50_ = 1.3 µM; SU‐DHL‐8, IC_50_ = 1.9 µM; OCI‐LY‐19, IC_50_ = 2.1 µM; FARAGE, IC_50_ = 3.9 µM).

To uncover the importance of LRRK2 protein in the growth of GCB DLBCL cell lines (OCI‐LY‐19 and WSU‐DLCL2), we then performed a silencing of LRRK2 by siRNAs. LRRK2 silencing affected the cell growth causing a reduction in cell growth after 48 h of silencing (Figure 4B–D).

We then analyzed total and phosphorylated GSK3β and AKT proteins, due to their possible association with the function of LRRK2 [5, 27, 28]. LRRK2 indeed directly phosphorylates AKT1 at Ser 473 [29].

**FIGURE 5:**
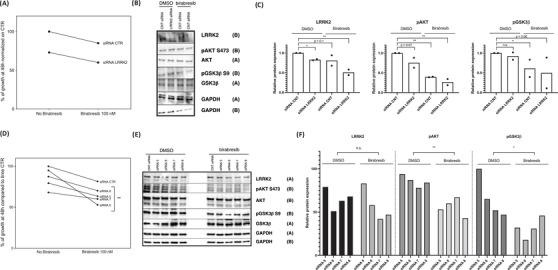
Birabresib improves the LRRK2 silencing effect in OCI‐LY‐19. (A) Viable cells, (B) immunoblot, representative of two replicates and (C) its relative quantification after treatment with a pool of siRNAs targeting LRRK2 in single or in combination with birabresib for 48 h. (D) Viable cells, (E) immunoblot, and (F) relative quantification after treatment with four single different siRNAs targeting LRRK2 in single or in combination with birabresib for 48 h. *p‐value ≤0.05; **p‐value ≤0.01; ***p‐value ≤0.001; n.s. = *p*‐value >0.1

LRRK2 silencing led to a downregulation of p‐AKT (S473) and p‐GSK3β (S9) levels (Figure 5 and Figure S6).

### 3.6 Genetic inhibition of LRRK2 synergizes with BET inhibitors in DLBCL cells

The effect on cell proliferation after LRRK2 silencing was increased when LRRK2 siRNAs were combined with the BET inhibitor birabresib. There was a beneficial effect on the proliferation between LRRK2 silencing and birabresib treatment in OCI‐LY‐19 (48 h) (Figure 5A) but not in the WSU‐DLCL2 (Figure S6A). To confirm this observation, we performed LRRK2 silencing with four individual siRNAs present in the pool. We obtained a consistent reduction of proliferation compared to control siRNAs that was increased when combined with birabresib (Figure 5D).

**FIGURE 6:**
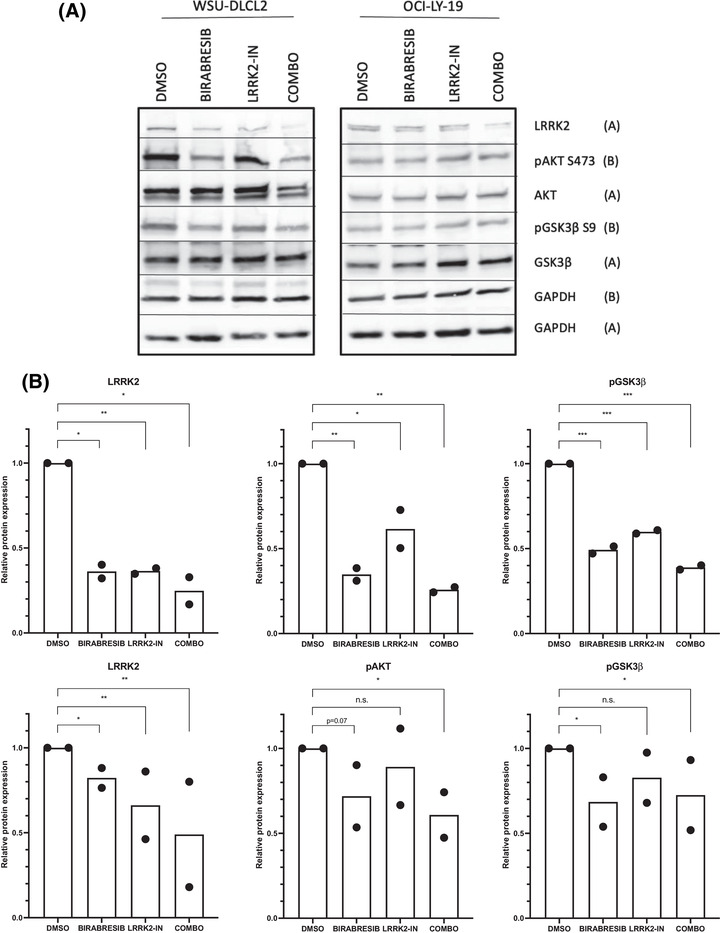
Birabresib and LRRK2‐IN induce a downregulation of p‐AKT (S473) and LRRK2. (A) Representatives immunoblot of two replicates. (B) Relative quantification of two replicates. WSU‐DLCL2 (top) and OCI‐LY‐19 (bottom) cell lines were treated with birabresib at 500 nM and LRRK2‐IN‐1 at 2 µM for 24 h. The expression of the proteins was normalized to the respective counterpart and to the housekeeping GAPDH. *p‐value ≤0.05; **p‐value ≤0.01; ***p‐value ≤0.001; n.s. = *p*‐value >0.1

Finally, when birabresib was added to the genetic silencing there was downregulation of p‐AKT (S473) and p‐GSK3β (S9) protein levels, stronger in OCI‐LY‐19 cell line (Figures 5B and 6C,E,F) than in WSU‐DLCL2 cell line (Figure S6B,C).

These data provided evidence of the novel role of LRRK2 in the sustaining pro‐survival pathways in lymphomas and validated the observed synergism of dual BET and LRRK2 inhibition.

### 3.7 Birabresib in combination with LRKK2‐IN‐1 affects LRRK2 expression

Based on the in vitro and in vivo antilymphoma activity of birabresib plus LRRK2‐IN‐1, we studied the mechanism of action of the drug combination in WSU‐DLCL2 and OCI‐LY‐19.

LRRK2 expression was affected by the single birabresib or LRRK2‐IN treatment, and the effect was higher in the combination (Figure 6). The downregulation of LRRK2 protein after birabresib single treatment can be explained by an observed lower BRD4 DNA binding, after JQ1, reported in publicly available chromatin immunoprecipitation (ChIP)‐sequencing dataset obtained from a GCB DLBCL cell line (SRP022129) (Figure S7). In addition, the downregulation of LRRK2 by LRRK2‐IN‐1 was in agreement with the reported protein destabilization and consequent proteasomal degradation induced by LRRK2 inhibitors [30]. Levels of both total and phosphorylated GSK3β and AKT were evaluated in single or combined treatment with birabresib and LRKK2‐IN‐1. The levels of p‐AKT (S473) and p‐GSK3β (S9) were downregulated by both compounds, while a slightly higher down‐regulation in both cell lines receiving the combination for 24 h was observed, compared to single treatments (Figure 6).

Based on the above‐demonstrated role of LRRK2 for the survival of lymphoma cells, the downregulation of LRRK2 by both LRRK2‐IN‐1 and birabresib can explain the observed synergism given by the combination of the two molecules.

## 4 DISCUSSION

A pharmacological screen with birabresib identified a short list of drugs that improved the antitumor activity of this BET inhibitor in lymphoma cell lines. Inhibitors of HDAC, mTOR, AKT, SRC, JAK, and LRRK2 showed the highest activity in combination with birabresib. While the inhibition of the first targets had already been reported as synergistic with BET inhibitors in lymphomas, less or no data were available for the combination with JAK and LRRK2 inhibitors, respectively [1]. Importantly, our data also identified LRRK2 as a novel putative target for lymphoma treatment with a prognostic role in DLBCL patients.

JAK inhibitors are approved for patients with myelofibrosis [31] and have shown signs of clinical activity especially in patients with Hodgkin lymphoma and T‐cell lymphomas [32, 33]. The combination with BET inhibitors is already being explored in myelofibrosis patients [34], and our data provide support for its extension to lymphoma patients.

SRC inhibitor dasatinib is already approved for patients with specific myeloid leukemias, and it is in clinical studies for other indications [35]. Multiple AKT inhibitors are being explored in the clinical setting [36]. Despite evidence supporting both SRC kinases and AKT as therapeutic targets in DLBCL [37, 38, 39, 40], the clinical activity of dasatinib and of the AKT inhibitor MK‐2206 as single agents has been limited [41]. Their use in combination seems more promising [37, 38, 39, 40]. Combinations with BET inhibitors might represent a class of compounds to be combined, as previously reported in MCL and Burkitt lymphoma models [9, 28]. The combination with the LRRK2 inhibitor LRRK2‐IN‐1 appeared relevant using both in vitro and in vivo models of GCB DLBCL.

LRRK2 (Leucine‐rich repeat kinase 2), also known as dardarin, is known for its involvement in some neurodegenerative diseases such as Parkinson's disease (PD) [42, 43]. It is a kinase involved in inflammation [26] and autophagy [25] and in the regulation of the WNT [23], MAPK [24], and MTOR [25] signaling pathways. Genome‐wide association studies have connected *LRRK2* locus polymorphisms with increased risk of immune diseases such as systemic lupus erythematosus [44], Crohn's disease [45], and inflammatory bowel disease [46] and with susceptibility to multibacillary leprosy [47]. Moreover, LRRK2 germline mutations are also linked with an overall increased risk of cancer, especially hormone‐related cancers and colorectal cancer [48]. In addition, LRRK2 promotes tumor cell growth and survival in papillary renal and in thyroid carcinomas [49] and proliferation together with metastatic capacity in intrahepatic cholangiocarcinoma cells [50]. LRRK2 has a dual activity as serine‐threonine kinase and as GTPase, and its biologic function is still largely undefined but apparently highly context dependent and involved in a wide range of signaling pathways [42, 43]. As serine‐threonine kinase, LRRK2 phosphorylates itself plus other already identified substrates, including AKT and GSK‐3β [29]. LRRK2 can be downstream to ATM, regulating the MDM2‐P53 axis [51], and directly phosphorylates AKT1 and enhances the kinase activity of GSK‐3β [5, 27, 28]. Our results represent the very first evidence for a possible role of LRRK2 in lymphomas, and for the inhibition of its kinase activity as potential therapeutic approach. The addition of LRRK2‐IN‐1 was synergistic with birabresib in all the six lymphoma cell lines, increasing apoptosis after BET inhibition, and the effect was in vivo validated using a GCB DLBCL xenograft. The synergism between LRRK2 inhibitors and BET inhibitors has been validated also combining LRRK2‐IN with a second BET inhibitor pelabresib and combining the LRRK2 inhibitors PF‐06447475 and GNE‐0877 with birabresib. Albeit part of the antitumor activity of LRRK2‐IN‐1 might be due to its binding to proliferating cell nuclear antigen or to the recently discovered affinity to BET proteins in addition to LRRK2 [52, 53], we showed that both the genetic and pharmacologic silencing of LRRK2 was toxic for lymphoma cells and gives an advantage to BET inhibitors. At the molecular level, the drug combination of LRRK2‐IN‐1 and birabresib compared to single treatments determined a downregulation of p‐AKT (S473) and LRRK2 itself in the two GCB DLBCL cell lines tested. The downregulation of LRRK2 after birabresib treatment can be explained by the demonstrated decreased BRD4 binding to the promoter of the *LRRK2* gene. The reduced LRRK2 protein levels after LRKK2‐IN‐1 are in line with the rapid protein destabilization and subsequent degradation already reported in multiple cellular models exposed to different LRRK2 inhibitors including the one we used [30]. Pharmacological inhibition of LRRK2 using small molecules targeting its kinase activity are entering the clinical evaluation for the treatment of Parkinson's disease [42], and promising safety data have been reported with the first‐in‐class compound DNL201 [54]. Our data suggest that these compounds might have a potential also for lymphoma patients.

In conclusion, we have identified a series of combinations that can improve the response to BET inhibitors in lymphomas, and we have identified LRRK2 as a gene essential for lymphomas and as a putative novel target for the development of antilymphoma agents.

## CONFLICT OF INTEREST

Emanuele Zucca: institutional research funds from Celgene, Roche and Janssen; advisory board fees from Celgene, Roche, Mei Pharma, Astra Zeneca and Celltrion Healthcare; travel grants from Abbvie and Gilead; expert statements provided to Gilead, Bristol‐Myers Squibb and MSD. Anastasios Stathis: institutional research funds from Bayer, ImmunoGen, Merck, Pfizer, Novartis, Roche, MEI Pharma, ADC‐Therapeutics; travel grant from AbbVie and PharmaMar. Eugenio Gaudio: currently employee of Helsinn; consultancy fees from Menarini, Cancer Research and Biotechnology (CRAB). Francesco Bertoni: institutional research funds from Acerta, ADC Therapeutics, Bayer AG, Cellestia, CTI Life Sciences, EMD Serono, Helsinn, ImmunoGen, Menarini Ricerche, NEOMED Therapeutics 1, Nordic Nanovector ASA, Oncology Therapeutic Development, PIQUR Therapeutics AG; consultancy fee from Helsinn, Menarini; expert statements provided to HTG; travel grants from Amgen, Astra Zeneca, Jazz Pharmaceuticals, PIQUR Therapeutics AG. Alberto J. Arribas received travel grant from Astra Zeneca and Luciano Cascione received travel grant from HTG. The other authors have no conflict of interest to disclose.

## Supporting information

Supporting Information

Supporting Information
