# Supplementary material for: Pharmacologic screen identifies active combinations with BET inhibitors and LRRK2 as a novel putative target in lymphoma
Source: EJHaem. 2022 Jul 27;3(3):764–74. doi: 10.1002/jha2.535 (PMC9422027; doi:10.1002/jha2.535)
Supplement: Supplementary file 1 — Supporting Information [file JHA2-3-764-s001.docx]

**Pharmacologic screening identifies active combinations with BET inhibitors and LRRK2 as a novel putative target in lymphoma**

Filippo Spriano ^1&^*, Giulio Sartori ^1&^, Chiara Tarantelli ^1^, Marilia Barreca ^1,2^, Gaetanina Golino ^1^, Andrea Rinaldi ^1^, Sara Napoli ^1^, Michele Mascia ^1^, Lorenzo Scalise ^1^, Alberto J. Arribas ^1,3^, Luciano Cascione ^1,3^, Emanuele Zucca ^1,4^, Anastasios Stathis ^4,5^, Eugenio Gaudio ^1^, Francesco Bertoni ^1,4*^

*^1^ Institute of Oncology Research, Faculty of Biomedical Sciences, USI, Bellinzona, Switzerland; ^2^* *Department of Biological, Chemical and Pharmaceutical Sciences and Technologies (STEBICEF), University of Palermo, Palermo, Italy; ^3^ SIB Swiss Institute of Bioinformatics, Lausanne, Switzerland; ^4^ Department of Oncology, Oncology Institute of Southern Switzerland, EOC, Bellinzona, Switzerland;* ^5^ *Faculty of Biomedical Sciences, USI, Lugano, Switzerland.*

^&^ co-first authors; *Corresponding authors

**Supplementary figures and table legend.**

**Supplementary Figure 1. Synergy parameters of potency and efficacy in LRRK2-IN-1 plus birabresib combination.** A) Efficacy of the combination. B), C) Potency of the combination.

Syn. Efficacy = synergistic efficacy, is the change in maximum efficacy compared to the most efficacious single agent. Syn. Potency = synergistic potency, is the magnitude of the change in the drug potency, owing to the presence of another drug. Efficacy > 1 = synergism, -1 < efficacy < 1 = additive, efficacy < -1 = antagonism. Potency > 0.5 = synergism, -0.5 < potency < 0.5 = additive, potency < -0.5 = antagonism.

**Supplementary Figure 2. Combinations of pelabresib with identified inhibitors in lymphoma cell lines.** Box-plots of the combination index (CI) values obtained in individual cell lines. Y-axis: CI values. In each box-plot, the line in the middle of the box represents the median CI value for the different concentrations combined. The box extends from the 25th to the 75th percentile (interquartile range, IQ); the whiskers extend to the upper and lower adjacent values (i.e., ±1.5 IQ); outside values have been omitted from the figure. CIs for pelabresib /LRRK2-IN-1 in OCI-LY-19 was not plotted due to median value > 3

**Supplementary Figure 3. Birabresib synergize with LRRK2 inhibitors PF-06447475 and GNE-0877.** Combinations between birabresib and LRRK2 inhibitors PF-06447475 and GNE-0877 in A) OCI-LY-19 and B) WSU-DLCL2 compared to predicted additivity curves. Predicted additivity calculated as (ratio of proliferating cells after birabresib) * (ratio of proliferating cells after LRRK2 inhibitor) at a specific concentration.

**Supplementary Figure 4. Birabresib combined with LRRK2-IN-1 treatment increase apoptosis induction.** Annexin V positive cells in A) WSU-DLCL2 and B) OCI-LY-19 treated with birabresib at 100nM or 500nM and LRRK2-IN-1 at 1 or 2μM, in single or combination for 72h. The percentage of Annexin V positive cells were normalized on CTR treated cells. C) Average percentage of annexin V positive cells in WSU-DLCL2 and OCI-LY-19. Experiment performed in at least two replicates.


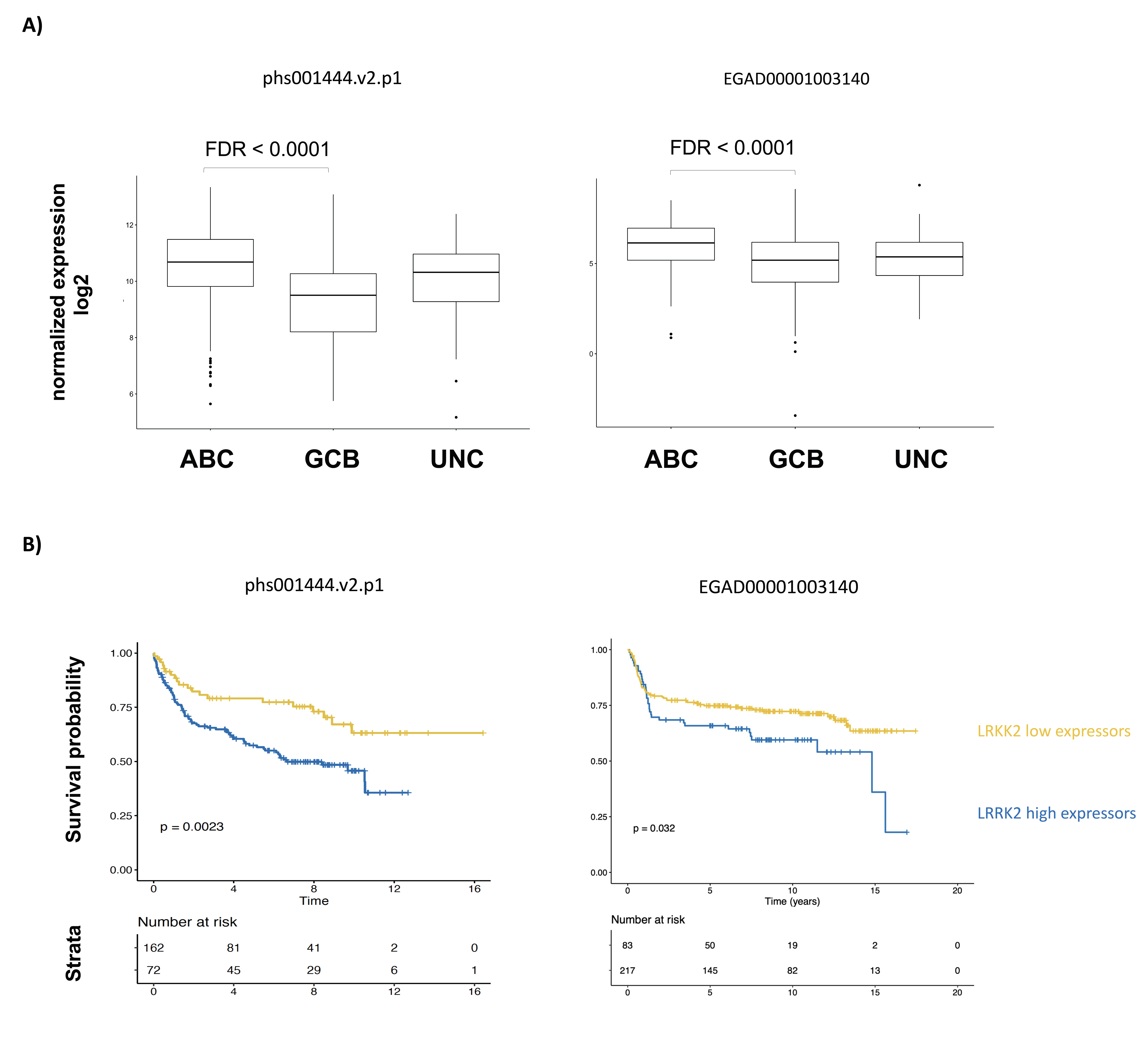
**Supplementary Figure 5. LRRK2 has a prognostic role in DLBCL clinical specimens.** A) LRRK2 expression in two datasets of DLBCL specimens divided by cell of origin. B) Survival curves for the high LRRK2 expressors group compared to the low LRRK2 expressors group in two datasets of DLBCL specimens. Raw data obtained from phs001444.v2.p1 ^1^ and EGAD00001003140 ^2^ .

**Supplementary Figure 6. Birabresib treatment combined with LRRK2 silencing in WSU-DLCL2.** A) Viable cells after treatment with birabresib or pool of siRNAs targeting LRRK2 in single and combination for 48h; representative experiment of two replicates. B) immunoblotting and C) relative quantification. * = p-value ≤ 0.05; ** = p-value ≤ 0.01; n.s. = p-value > 0.1.


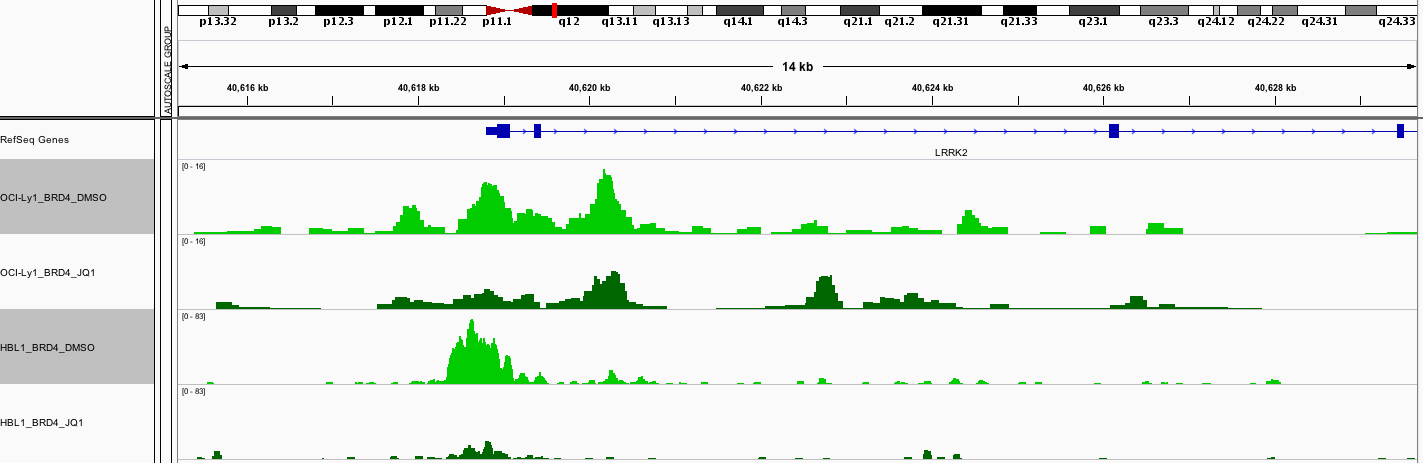


**Supplementary Figure 7. BRD4 DNA binding in the promoter region of LRRK2 after BET inhibitor** **treatment.** BRD4 DNA binding as assessed by ChIP-Seq (SRP022129) in two DLBCL cell lines (OCI-LY-1 and HBL1) after treatment with DMSO as control (light green) and JQ1 as BET inhibitor (dark green); the LRRK2 gene is shown in blue.

**References**

1. Schmitz R, Wright GW, Huang DW, Johnson CA, Phelan JD, Wang JQ, et al. Genetics and Pathogenesis of Diffuse Large B-Cell Lymphoma. N Engl J Med. 2018;378(15):1396-1407.

2. Ennishi D, Mottok A, Ben-Neriah S, Shulha HP, Farinha P, Chan FC, et al. Genetic profiling of MYC and BCL2 in diffuse large B-cell lymphoma determines cell-of-origin-specific clinical impact. Blood. 2017;129(20):2760-2770.

**Supplementary Table 1. Percentage of proliferating cells and fold changes between single treatments and combinations.** WSU-DLCL2 and OCI-LY-19 GCB DLBCL cell lines exposed to birabresib (100nM) or library compounds (20nM, 1000nM).
